# Anatomical Predictors of Endovascular Technical Success in Chronic Limb-Threatening Ischemia: The Role of GLASS and Diabetes

**DOI:** 10.3390/medsci14060614

**Published:** 2026-09-29

**Authors:** Anca Dumitrescu-Bordianu, Livia Genoveva Baroi, Radu Florin Popa, Lucian Iacob, Bogdan Caba, Paula Cristina Morariu, Maria Mihaela Godun, Ioana-Cezara Caba, Florin Petrariu, Anton Knieling, Bogdan Huzum, Alin Horatiu Nedelcu, Mariana Floria

**Affiliations:** 1 Department of Vascular Surgery, Grigore T. Popa University of Medicine and Pharmacy, 700115 Iasi, Romania; bordianu.anca@d.umfiasi.ro (A.D.-B.); radu.popa@umfiasi.ro (R.F.P.); luciiacob@gmail.com (L.I.); 2 Faculty of Medical Bioengineering, Grigore T. Popa University of Medicine and Pharmacy, 700115 Iasi, Romania; 3 Department of Internal Medicine, Grigore T. Popa University of Medicine and Pharmacy, 700115 Iasi, Romania; morariu.paula-cristina@email.umfiasi.ro (P.C.M.); godun.maria-mihaela@d.umfiasi.ro (M.M.G.); floria.mariana@umfiasi.ro (M.F.); 4 Faculty of Pharmacy, Grigore T. Popa University of Medicine and Pharmacy, 700115 Iasi, Romania; ioana-cezara.caba@umfiasi.ro; 5 Department of Preventive Medicine and Interdisciplinarity, Faculty of Medicine, Grigore T. Popa University of Medicine and Pharmacy, 700115 Iasi, Romania; florin.petrariu@umfiasi.ro; 6 Discipline of Forensic Medicine, Faculty of Medicine, Grigore T. Popa University of Medicine and Pharmacy, 700115 Iasi, Romania; anton.knieling@umfiasi.ro; 7 Department of Surgical Sciences II, Discipline of Orthopedics and Traumatology, Grigore T. Popa University of Medicine and Pharmacy, 700115 Iasi, Romania; bogdan.huzum@umfiasi.ro; 8 Department of Morpho-Functional Sciences I, Discipline of Anatomy and Embryology, Grigore T. Popa University of Medicine and Pharmacy, 700115 Iasi, Romania; alin.nedelcu@umfiasi.ro

**Keywords:** chronic limb-threatening ischemia, GLASS classification, endovascular revascularization, technical success, angiographic complexity, diabetes mellitus

## Abstract

**Background:** Chronic limb-threatening ischemia (CLTI) is associated with high rates of major adverse limb events and mortality, particularly in patients with diabetes mellitus (DM). The Global Limb Anatomic Staging System (GLASS) standardizes anatomical assessment and supports revascularization planning. This study evaluated the association of anatomical complexity and diabetes with immediate technical success after infrainguinal endovascular treatment. **Methods:** This single-center retrospective study included 136 patients undergoing infrainguinal endovascular procedures for CLTI between May 2021 and May 2023, stratified into diabetic (*n* = 85) and non-diabetic (*n* = 51) groups. Anatomical complexity was assessed using GLASS. Technical success was defined as restoration of inline flow to the foot. Logistic regression and receiver operating characteristic analyses were performed. **Results:** Technical success was achieved in 74.3% of procedures, without significant differences between diabetic and non-diabetic patients (75.3% vs. 72.5%, *p* = 0.723). Success declined progressively from 86.3% in GLASS stage I to 75.5% in stage II and 53.1% in stage III (*p* = 0.003; trend *p* = 0.001). In multivariable analysis, GLASS FP (femoropopliteal) grade ≥2 (adjusted OR 8.068, 95% CI 3.018–21.562; *p* < 0.001) and tibioperoneal trunk occlusion (adjusted OR 4.874, 95% CI 1.271–18.687; *p* = 0.021) were independently associated with technical failure, whereas diabetes was not. The model showed good discrimination (AUC 0.820, 95% CI 0.744–0.897; *p* < 0.001). **Conclusions:** Anatomical complexity was associated with immediate technical success in CLTI, whereas no independent association with diabetes was detected in this cohort. GLASS may provide clinically relevant preprocedural risk stratification and support endovascular planning.

## 1. Introduction

Chronic limb-threatening ischemia (CLTI) represents the most advanced stage of peripheral arterial disease. It is associated with high rates of limb loss and mortality despite ongoing advances in revascularization strategies [1,2]. Accurate risk stratification and appropriate procedural planning remain essential for improving outcomes in this vulnerable patient population. Chronic total occlusions represent a major technical barrier in peripheral interventions, as successful revascularization depends on recanalizing the lesion before delivering therapy [3,4]. Over the past few years, there has been a progressive shift from purely clinical classifications towards integrated models that incorporate anatomical assessment [5,6]. The Global Limb Anatomic Staging System (GLASS), introduced within the Global Vascular Guidelines framework, provides a standardized way to grade angiographic lesions [1,7,8,9]. By structuring disease severity across femoropopliteal and infrapopliteal segments, GLASS provides a reproducible framework for stratifying procedural complexity and enabling practitioners to judge expected technical feasibility. Evidence supports the prognostic relevance of GLASS staging in CLTI. In a systematic review and meta-analysis including more than 2000 patients, Shirasu et al. found that a higher GLASS stage was associated with worse limb-related outcomes and higher procedural failure rates, with immediate technical failure rising from 3.9% in stage 1 to nearly 28% in stage 3 following endovascular treatment [10]. A recent meta-analysis by Bontinis et al. further supported the prognostic value of GLASS, showing poorer procedural and limb-related outcomes with increasing anatomical complexity [11]. These findings are supported by contemporary cohort data. Luan et al. showed that higher GLASS stages were associated with a progressive reduction in immediate technical success, along with higher rates of reintervention and major adverse limb events during long-term follow-up [12,13]. An important emerging concept is the role of segmental anatomical complexity. Recent studies have shown that the prognostic impact of GLASS is not uniform across the infrainguinal segment, with below-the-knee and inframalleolar disease profoundly affecting both procedural and clinical outcomes. In patients with isolated infrapopliteal lesions, higher GLASS stages have been associated with reduced procedural success and less favorable perioperative results [14]. The introduction of the inframalleolar modifier, identifying the quality of foot blood flow, has provided additional prognostic prediction and has shown a significant association with limb salvage and wound healing following endovascular revascularization [15]. More recently, advanced segmental patterns, such as the GLASS P2 modifier, have been proposed to recognize “no-option” anatomical configurations, further emphasizing the importance of detailed anatomical pre-procedural work-up [16].

Nevertheless, clinical classification systems such as the Society for Vascular Surgery Wound, Ischemia, and Foot Infection (WIfI) score are widely used to stratify limb-related risk in patients with CLTI. In a large-scale study, higher WIfI stages were associated with a marked increase in the risk of major amputation, with hazard ratios exceeding 2.0 per stage increment and even higher when composite scores were applied [17,18]. However, these systems primarily reflect the severity of tissue loss and infection rather than the anatomical characteristics of arterial disease. As a result, their ability to predict procedural feasibility and immediate technical success remains limited, particularly in endovascular interventions, where lesion morphology plays a more important role.

Against this background, the present study addresses this gap by evaluating immediate technical success in a real-world Eastern European cohort of patients with infrainguinal CLTI, with a direct comparison between diabetic and non-diabetic patients and a segment-specific assessment of the femoropopliteal and infrapopliteal components of the GLASS classification.

At our institution, we have used open surgical revascularization to manage CLTI for more than two decades, and we introduced an endovascular treatment program in 2021. This transition provided the rationale for evaluating whether anatomical complexity, as assessed by the GLASS classification, could support a standardized approach to endovascular treatment and help identify patients at increased risk of immediate technical failure.

This study aimed to evaluate the prognostic performance of the GLASS classification in predicting technical success in patients with infrainguinal CLTI undergoing endovascular revascularization [19]. We also sought to determine whether diabetes mellitus (DM) independently influences procedural outcomes or primarily modifies anatomical disease severity.

## 2. Materials and Methods

This retrospective, single-center study was conducted in the Vascular Surgery Department of “St. Spiridon” Emergency Clinical Hospital, Iași, Romania. We screened all consecutive patients who underwent lower limb revascularization between May 2021 and May 2023 for eligibility. We individualized the choice between endovascular and open revascularization based on lesion anatomy, patient comorbidities, operative risk, conduit availability, and institutional expertise. Patients aged ≥18 years with angiographically confirmed peripheral arterial disease and clinical evidence of CLTI (generally corresponding to advanced Fontaine stages III–IV) who underwent infrainguinal endovascular revascularization were included. We staged clinical limb severity using the WIfI classification, which we systematically collected for the study cohort.

Exclusion criteria were: (1) primary open surgical revascularization (open bypass) or any treatment other than endovascular revascularization, as the study aimed to evaluate predictors of immediate technical success after endovascular treatment; (2) aorto-iliac disease, as the study focused on infrainguinal CLTI; and (3) incomplete clinical, angiographic, or GLASS data. Figure 1 summarizes patient selection. We divided patients into two groups by diabetes status: DM and non-diabetic (NDM). We collected baseline demographic and clinical characteristics, including cardiovascular comorbidities, from hospital records and compared them between the DM and NDM groups.

All procedures were performed in an angiography suite under fluoroscopic guidance using standard endovascular techniques. Vascular access was obtained percutaneously via the common femoral artery or, in selected cases, via alternative access, including distal or direct puncture techniques. We used either a contralateral retrograde approach or an ipsilateral antegrade approach, depending on lesion location and anatomical considerations. Diagnostic angiography was performed using digital subtraction angiography to assess lesion morphology and distal runoff. Systemic anticoagulation with unfractionated heparin was administered intra-procedurally according to standard institutional protocols. Lesions were primarily treated with plain balloon angioplasty. Prolonged inflation times were routinely used, and adjunctive techniques, such as repeated dilatation, were employed when necessary. Stent implantation was performed selectively in cases of significant residual stenosis, flow-limiting dissection, or elastic recoil. Revascularization strategy followed a pragmatic “easiest-first” approach, prioritizing the technically most feasible target vessel rather than strict angiosome-guided revascularization.

Technical success was defined as restoration of inline flow to the foot with <30% residual stenosis on completion angiography. Technical failure was defined as the inability to achieve inline flow to the foot and/or the presence of residual stenosis ≥30% despite endovascular treatment.

Major amputation and mortality were recorded as in-hospital outcomes during the index hospitalization.

We classified lesions using the GLASS, including both femoropopliteal and infrapopliteal segments, according to the Global Vascular Guidelines. The femoropopliteal (FP) and infrapopliteal (IP) components were graded separately from 0 to 4 according to segment-specific anatomical complexity. The FP grade reflects the severity and extent of disease along the superficial femoral and popliteal arterial segments, whereas the IP grade reflects disease involving the tibial and peroneal arteries along the target arterial path, with increasing grades representing progressively greater anatomical complexity. These segmental grades were integrated according to the GLASS framework to assign an overall GLASS stage (I–III), reflecting the anatomical complexity of the target arterial path. GLASS staging was performed retrospectively. The investigator performing GLASS assessment was not formally blinded to the immediate procedural outcome, as the complete angiographic sequence was available during retrospective assessment. Secondary data included peri-procedural findings and lesion characteristics.

Patients undergoing stenting or infrapopliteal PTA received dual antiplatelet therapy with acetylsalicylic acid (aspirin) 75 mg once daily and clopidogrel 75 mg once daily for up to 6 weeks. In patients not previously receiving antiplatelet therapy, loading doses of 300 mg acetylsalicylic acid and 300 mg clopidogrel were administered.

Table 1 and Table 2 summarise the baseline clinical characteristics of the study population according to diabetic status and the distribution of stent implantation between diabetic and non-diabetic patients, respectively.

Statistical analysis was performed using SPSS (version 29, IBM Corp., Armonk, NY, USA). Continuous variables were expressed as mean ± standard deviation or median and IQR, and categorical variables were expressed as frequencies and percentages. We checked the normality of continuous variables using the Shapiro–Wilk test; because the results showed asymmetrical distributions, we performed group comparisons using the Mann–Whitney U test. For categorical variables, we compared groups using the Pearson Chi-square test and applied the Fisher correction when needed. We also evaluated the association between overall GLASS stage and immediate technical outcome using the linear-by-linear association test to assess trends across increasing GLASS stages. We performed univariable binary logistic regression to estimate the association between age and technical failure. Crude odds ratios for the remaining clinical and anatomical variables were estimated in the same way (Table 3 and Table 4, Figure 2). Multivariable binary logistic regression analysis was performed to identify the predictors of technical failure (Enter method), without automated variable selection (e.g., stepwise procedures). Predictors’ multicollinearity was assessed using tolerance and variance inflation factors (VIF). Model fit was assessed with the omnibus likelihood-ratio test, the Hosmer–Lemeshow goodness-of-fit test and Nagelkerke R^2^. Internal stability of the estimates was examined with bias-corrected and accelerated (BCa) bootstrap 95% CIs based on 1000 resamples. Results were expressed as odds ratios (OR) with 95% confidence intervals (CI). We evaluated the predictive model’s discriminative performance using receiver operating characteristic (ROC) curve analysis and calculated the area under the curve (AUC). We considered *p* < 0.05 statistically significant.

## 3. Results

### Study Population

A total of 201 patients underwent lower limb revascularization during the study period. After applying exclusion criteria, we included 136 patients with CLTI with complete clinical, imaging, and GLASS data in the final analysis. Of these, 85 patients were in the DM group and 51 patients in the NDM group. Figure 1 summarizes patient selection. Patients with aorto-iliac disease were excluded because the study focused on revascularization for CLTI of the infrainguinal region.

Baseline demographic and clinical characteristics according to diabetic status are presented in Table 1. Mean age was similar between diabetic and non-diabetic patients (69.89 ± 8.57 vs. 69.78 ± 11.38 years, *p* = 0.737). Urban residence was more frequent among patients with diabetes (72.9% vs. 49.0%, *p* = 0.005). No statistically significant differences were observed between the groups for the other baseline comorbidities.

**Table 1:** Baseline clinical characteristics according to diabetic status.

Variable		Total (*n* = 136)	Non-Diabetic (*n* = 51)	Diabetic (*n* = 85)	*p*-Value
Male sex		97 (71.3%)	39 (76.5%)	58 (68.2%)	0.304
Urban environment		87 (64.0%)	25 (49.0%)	62 (72.9%)	0.005
Age (years)	Mean ± SD	69.85 ± 9.674	69.78 ± 11.376	69.89 ± 8.566	0.737
	Median	70.00	70.00	70.00	
	IQR	(66.00 ÷ 75.00)	(67.00 ÷ 77.00)	(65.50 ÷ 75.00)	
Hypertension		111 (81.6%)	38 (74.5%)	73 (85.9%)	0.097
Obesity		17 (12.5%)	4 (7.8%)	13 (15.3%)	0.203
Heart failure		41 (30.1%)	17 (33.3%)	24 (28.2%)	0.531
Atrial fibrillation		31 (22.8%)	15 (29.4%)	16 (18.8%)	0.154
Previous myocardial infarction		9 (6.6%)	2 (3.9%)	7 (8.2%)	0.483
Chronic coronary syndrome		25 (18.4%)	8 (15.7%)	17 (20.0%)	0.530
Hypertrophic cardiomyopathy		6 (4.4%)	1 (2.0%)	5 (5.9%)	0.410
Dilated cardiomyopathy		8 (5.9%)	4 (7.8%)	4 (4.7%)	0.473
Chronic kidney disease		23 (16.9%)	7 (13.7%)	16 (18.8%)	0.443
Stroke history		12 (8.9%)	2 (3.9%)	10 (11.9%)	0.210

Values are expressed as a number (percentage). Comparisons between groups were performed using the Pearson chi-square test or Fisher’s exact test, as appropriate. Fisher’s exact test was used for previous myocardial infarction and previous stroke. We considered *p* < 0.05 statistically significant.

Selective stent implantation was required in 31 patients (22.8%), without significant differences between diabetic and non-diabetic patients (23.5% vs. 21.6%, *p* = 0.792). Most patients received a single stent, whereas implantation of multiple stents was uncommon (Table 2).

**Table 2:** Distribution of stent implantation according to diabetes status.

Variable	Non-Diabetic (*n* = 51)	Diabetic (*n* = 85)	*p*-Value
Any stent implantation, *n* (%)	11 (21.6)	20 (23.5)	0.792
One stent, *n* (%)	8 (15.7)	14 (16.5)	
Two stents, *n* (%)	2 (3.9)	2 (2.4)	
Three stents, *n* (%)	1 (2.0)	4 (4.7)	

Values are expressed as a number (percentage). Comparisons between groups were performed using the Pearson chi-square test. No statistically significant differences were observed between diabetic and non-diabetic patients. Note: Of the 31 procedures involving stent implantation, stents were placed in the superficial femoral artery in 30 cases (96.8%) and at the origin of the anterior tibial artery in one case (3.2%).

Primary outcome: technical success was achieved in 101 of 136 procedures (74.3%), while technical failure occurred in 35 cases (25.7%). Failure was mainly due to inability to cross the target lesion (wire- or balloon-uncrossable) because of severe calcification, chronic total occlusion, or proximal vessel disease. Success rates were similar between diabetic and non-diabetic patients (75.3% vs. 72.5%, *p* = 0.723).

In the comparative study of the sample’s baseline characteristics amongst the patients with technical failure and those with technical success, the following results were obtained: the gender distribution was similar within the two investigated samples; the number of patients from rural areas was slightly higher among those with technical failure than among the others (42.9% vs. 33.7%), and the patients with technical failure were on average almost two years older, but none of these differences reached statistical significance. Hypertension was significantly less frequent among patients with technical failure (65.7% vs. 87.1% of patients with technical success), so its presence appeared inversely associated with failure. No other statistically significant differences were observed for the remaining comorbidities investigated. It may nevertheless be noted that obesity (14.3% vs. 11.9%), chronic kidney disease (17.1% vs. 16.8%) and previous stroke (11.4% vs. 7.9%) were slightly more frequent in the technical failure group than in the other, although the differences were rather small. The proportion of patients with DM was also similar between the two groups: 60.0% in the technical failure group compared with 63.4% in the technical success group (Table 3).

**Table 3:** Comparative study of the baseline clinical characteristics according to the technical outcome (global).

		Technical Outcome		*p*-Value	OR (95% C.I.)
		Failure (*n* = 35)	Success (*n* = 101)		
Male sex		25 (71.4%)	72 (71.3%)	0.987	1.007 (0.430 ÷ 2.357)
Urban environment		20 (57.1%)	67 (66.3%)	0.329	0.677 (0.308 ÷ 1.486)
Age	Mean ± SD	71.29 ± 8.953	69.36 ± 9.906	0.178	1.022 (0.980 ÷ 1.065)
	Median	72.00	70.00		
	IQR	(67.00 ÷ 77.00)	(64.00 ÷ 74.00)		
Diabetes Mellitus (DM)		21 (60.0%)	64 (63.4%)	0.723	0.867 (0.394 ÷ 1.907)
Hypertension		23 (65.7%)	88 (87.1%)	0.005	0.283 (0.114 ÷ 0.703)
Obesity		5 (14.3%)	12 (11.9%)	0.768	1.236 (0.402 ÷ 3.797)
Heart failure		9 (25.7%)	32 (31.7%)	0.507	0.746 (0.314 ÷ 1.775)
Atrial fibrillation		8 (22.9%)	23 (22.8%)	0.992	1.005 (0.402 ÷ 2.511)
Previous Myocardial Infarction		2 (5.7%)	7 (6.9%)	1.000	0.814 (0.161 ÷ 4.116)
Chronic Coronary Syndrome		6 (17.1%)	19 (18.8%)	0.826	0.893 (0.325 ÷ 2.454)
Hypertrophic cardiomyopathy		1 (2.9%)	5 (5.0%)	1.000	0.565 (0.064 ÷ 5.008)
Dilated cardiomyopathy		2 (5.7%)	6 (5.9%)	1.000	0.960 (0.185 ÷ 4.990)
Chronic Kidney Disease		6 (17.1%)	17 (16.8%)	0.966	1.022 (0.368 ÷ 2.841)
Stroke history		4 (11.4%)	8 (7.9%)	0.505	1.500 (0.422 ÷ 5.326)

Age was compared between groups using the Mann–Whitney U test, whereas categorical variables were compared using the Pearson chi-square test or Fisher’s exact test when necessary. We considered *p* < 0.01 statistically highly significant.

Regarding distal arterial anatomy, we identified occlusions in the anterior tibial artery in 55 patients (40.4%), the posterior tibial artery in 64 (47.1%), the tibioperoneal trunk in 19 (14.0%), and the fibular artery in 35 (25.7%), providing additional characterization of the infrapopliteal disease distribution.

We also compared the anatomical characteristics of patients with technical failure and those with technical success (Table 4).

WIfI stage did not differ significantly between patients with technical failure and those with technical success (*p* = 0.233). WIfI stage 1 was recorded in 51.4% vs. 44.6% of patients, and stages 3–4 in 31.4% vs. 25.7%, respectively.

Stents were present in 27.7% of patients with technical success compared with only 8.6% of those with technical failure, a statistically significant difference; the presence of stents reduces the odds of technical failure by 75.6%. Stenotic plaques were present in 76.2% of patients with technical success compared with only 51.4% of those with technical failure—again a highly significant difference—the presence of stenotic plaques being associated with 67.0% lower odds of failure. Stenotic plaques greater than 25% were present in 68.3% of patients with technical success compared with only 45.7% with technical failure; stenotic plaques greater than 50% were present in 50.5% of patients with technical success compared with 40.0% with technical failure. Occlusion was observed in almost all patients with technical failure (97.1%), but also in a large proportion of those with technical success (85.1%); the difference was close to statistical significance.

For superficial femoral artery (SFA) lesions, we also found statistically significant overall differences. Among patients with technical failure, slightly more than half had an occlusion (54.3%), and 28.6% had plaques with stenosis greater than 50%, whereas among those with technical success, only 28.7% had an occlusion and 26.7% had plaques with stenosis greater than 50%. Accordingly, the odds of technical failure were higher in limbs with SFA stenosis >25% (including occlusions; 91.4% vs. 67.3%; OR 5.176, stenosis >50% (including occlusions; 82.9% vs. 55.4%; OR 3.884), and occlusion alone (54.3% vs. 28.7%; OR 2.948). Statistically significant differences were also found in the overall cohort for popliteal artery (POP) lesions. Among patients with technical success, 61.4% had no popliteal lesions, 13.9% had plaques with stenosis greater than 50% and only 7.9% had an occlusion; in contrast, among patients with technical failure, although 54.3% had no lesions, 11.4% had plaques with stenosis greater than 50% and 28.6% had an occlusion; cumulatively, the odds of technical failure were higher in limbs with POP stenosis >50% (including occlusions; 40.0% vs. 21.8%; OR 2.394, 95% CI 1.049–5.462) and in those with occlusion alone (28.6% vs. 7.9%; OR 4.650, 95% CI 1.661–13.015). As for the anterior tibial artery (ATA), no statistically significant differences were found between patients with technical success and those with technical failure. For the posterior tibial artery (PTA), in the overall cohort, 57.1% of patients with technical failure had no lesions, and 37.1% had an occlusion, whereas among those with technical success, 34.7% had no lesions and 50.5% had an occlusion—a difference close to the threshold of statistical significance, again indicating a slightly less severe involvement of the PTA in patients with technical failure than in the others.

In the tibioperoneal trunk (TPT), statistically significant differences in lesion pattern were found overall between patients with technical failure and those with technical success. Among patients with technical failure, 28.6% had an occlusion compared with only 8.9% of those with technical success; occlusion thus increased the odds of failure 4.089-fold, whereas plaques with stenosis greater than 50% were inversely associated with failure.

GLASS grade in the femoropopliteal (FP) segment—the assessment showed highly significant differences between patients with technical failure and those with technical success. In the overall cohort, almost half of the patients with technical failure had GLASS FP grade 3 or 4 (45.8%), compared with only 11.9% of those with technical success; a GLASS FP grade of 4 increased the odds of failure 5.689-fold, a grade ≥3 increased them 6.246-fold, and a grade ≥2 increased them 6.229-fold.

GLASS grade in the infrapopliteal (IP) segment also differed significantly between patients with technical failure and those with technical success: in the overall cohort, 28.6% of patients with technical failure had GLASS IP grade 4, compared with only 8.9% of those with technical success, so that IP grade 4 increased the odds of failure 4.089-fold.

Overall, 42.9% of patients with technical failure had GLASS stage 3, compared with 16.8% of those with technical success—a highly significant difference, GLASS stage 3 increasing the odds of failure 3.706-fold and GLASS stage ≥ 2 increasing them 3.088-fold.

**Table 4:** Comparative study of the anatomical characteristics according to the technical outcome (global).

		Technical Outcome		*p*-Value	OR (95% C.I.)
		Failure (*n* = 35)	Success (*n* = 101)		
WIfI stage	WIfI 1	18 (51.4%)	45 (44.6%)	0.233	-
	WIfI 2	6 (17.1%)	30 (29.7%)		
	WIfI 3	10 (28.6%)	18 (17.8%)		
	WIfI 4	1 (2.9%)	8 (7.9%)		
Stents		3 (8.6%)	28 (27.7%)	0.020 *	0.244 (0.069 ÷ 0.863)
Stenotic plaques		18 (51.4%)	77 (76.2%)	0.006 **	0.330 (0.147 ÷ 0.739)
Stenotic plaques > 25%		16 (45.7%)	69 (68.3%)	0.017 *	0.391 (0.178 ÷ 0.857)
Stenotic plaques > 50%		14 (40.0%)	51 (50.5%)	0.284	0.654 (0.299 ÷ 1.427)
Occlusion		34 (97.1%)	86 (85.1%)	0.070	5.930 (0.754 ÷ 46.660)
SFA	no changes	2 (5.7%)	21 (20.8%)	0.029 *	stenotic plaques > 25%:
	stenotic plaques < 25%	1 (2.9%)	12 (11.9%)		5.176 (1.477 ÷ 18.147)
	stenotic plaques 25–50%	3 (8.6%)	12 (11.9%)		stenotic plaques > 50%:
	stenotic plaques > 50%	10 (28.6%)	27 (26.7%)		3.884 (1.483 ÷ 10.170)
	occlusion	19 (54.3%)	29 (28.7%)		occlusion:
					2.948 (1.335 ÷ 6.513)
POP	no changes	19 (54.3%)	62 (61.4%)	0.026 *	stenotic plaques > 50%:
	stenotic plaques < 25%	1 (2.9%)	9 (8.9%)		2.394 (1.049 ÷ 5.462)
	stenotic plaques 25–50%	1 (2.9%)	8 (7.9%)		occlusion:
	stenotic plaques > 50%	4 (11.4%)	14 (13.9%)		4.650 (1.661 ÷ 13.015)
	occlusion	10 (28.6%)	8 (7.9%)		
ATA	no changes	20 (57.1%)	46 (45.5%)	0.493	-
	stenotic plaques 25–50%	0 (0.0%)	3 (3.0%)		
	stenotic plaques > 50%	2 (5.7%)	10 (9.9%)		
	occlusion	13 (37.1%)	42 (41.6%)		
PTA	no changes	20 (57.1%)	35 (34.7%)	0.090	stenotic plaques > 25%:
	stenotic plaques 25–50%	0 (0.0%)	5 (5.0%)		0.398 (0.181 ÷ 0.872)
	stenotic plaques > 50%	2 (5.7%)	10 (9.9%)		
	occlusion	13 (37.1%)	51 (50.5%)		
TPT	no changes	23 (65.7%)	79 (78.2%)	0.020 *	stenotic plaques > 50%:
	stenotic plaques 25–50%	0 (0.0%)	6 (5.9%)		0.398 (0.181 ÷ 0.872)
	stenotic plaques > 50%	2 (5.7%)	7 (6.9%)		occlusion:
	occlusion	10 (28.6%)	9 (8.9%)		4.089 (1.499 ÷ 11.150)
FA (fibular artery)	no changes	22 (62.9%)	71 (70.3%)	0.147	-
	stenotic plaques 25–50%	0 (0.0%)	4 (4.0%)		
	stenotic plaques > 50%	0 (0.0%)	4 (4.0%)		
	occlusion	13 (37.1%)	22 (21.8%)		
GLASS	0	3 (8.6%)	21 (20.8%)	<0.001 **	Glass grade FP ≥ 2
Grade FP	1	6 (17.1%)	48 (47.5%)		6.229 (2.620 ÷ 14.812)
	2	10 (28.6%)	20 (19.8%)		Glass grade FP ≥ 3
	3	8 (22.9%)	7 (6.9%)		6.246 (2.546 ÷ 15.324)
	4	8 (22.9%)	5 (5.0%)		Glass grade FP = 4
					5.689 (1.720 ÷ 18.815)
GLASS	0	8 (22.9%)	13 (12.9%)	0.012 *	Glass grade IP = 4
Grade IP	1	10 (28.6%)	39 (38.6%)		4.089 (1.499 ÷ 11.150)
	2	3 (8.6%)	23 (22.8%)		
	3	4 (11.4%)	17 (16.8%)		
	4	10 (28.6%)	9 (8.9%)		
GLASS	STAGE 1	7 (20.0%)	44 (43.6%)	0.003 **	Glass grade ≥ 2
	STAGE 2	13 (37.1%)	40 (39.6%)		3.088 (1.234 ÷ 7.724)
	STAGE 3	15 (42.9%)	17 (16.8%)		Glass grade = 3
					3.706 (1.586 ÷ 8.657)

Categorical variables were compared using the Pearson chi-square test or Fisher’s exact test when necessary. A *p*-value < 0.05 * was considered statistically significant; a *p*-value < 0.01 ** was considered statistically highly significant. Odds ratios for SFA and POP refer to cumulative categories (e.g., stenosis > 50% includes occlusions), each compared with all remaining limbs.

Overall, GLASS stage was significantly associated with immediate technical outcome (Figure 2). Technical success decreased progressively with increasing anatomical complexity, from 86.3% in GLASS stage I to 75.5% in stage II and 53.1% in stage III, whereas technical failure increased from 13.7% to 24.5% and 46.9%, respectively. The association showed also a significant linear trend across increasing GLASS stages (Linear-by-Linear Association, *p* = 0.001).

**Figure 2:**
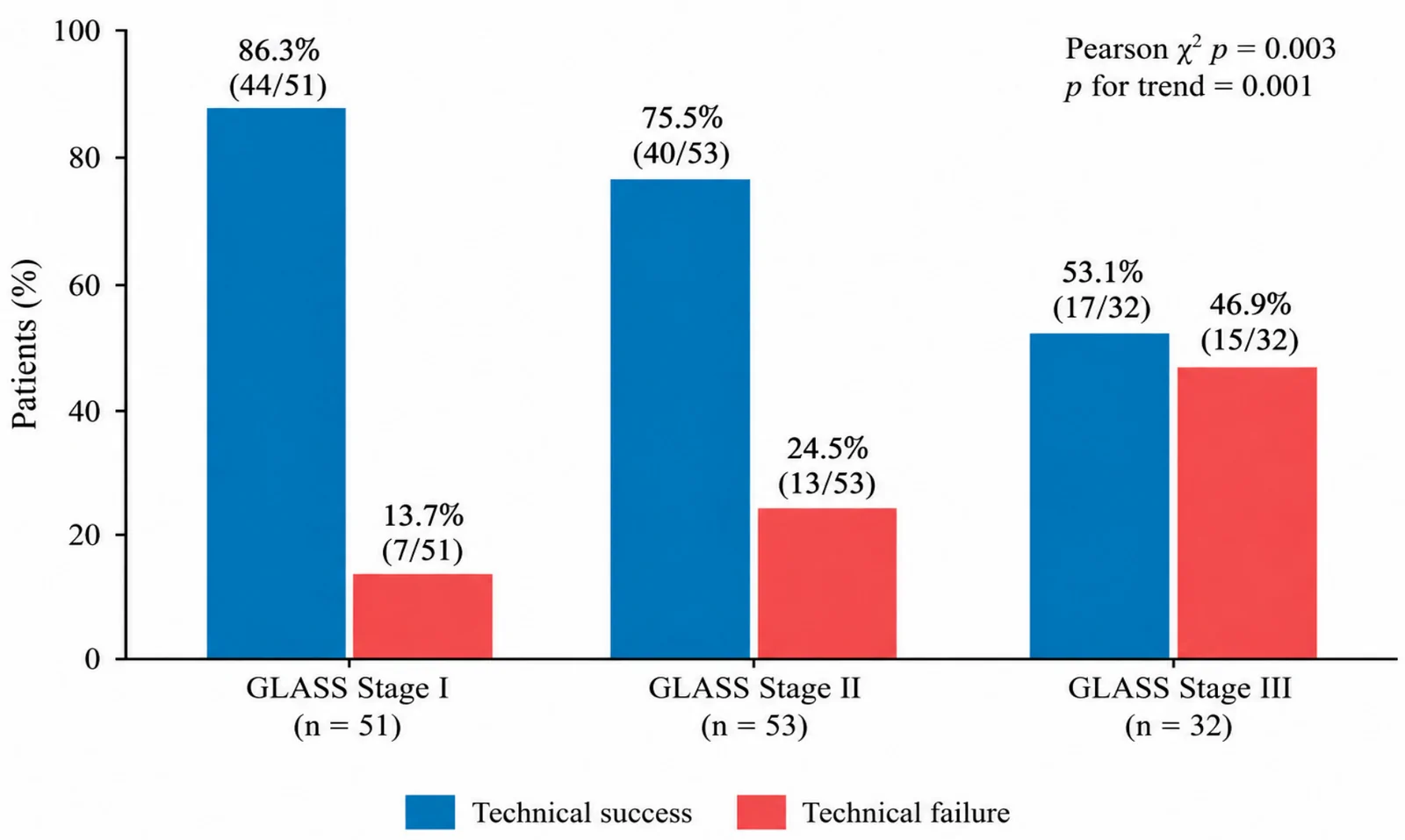
Immediate technical outcome according to overall GLASS stage. Technical success decreased progressively with increasing GLASS stage (Pearson χ^2^, *p* = 0.003; Linear-by-Linear Association, *p* = 0.001).

In this way, the set of risk factors for technical failure was identified in the overall cohort (Table 3 and Table 4; Figure 3). From these, the candidate variables for the multivariable binary logistic regression model were selected. Given that technical failure was recorded in 35 cases, and following the rule of thumb that a viable model should contain approximately 10 events per predictor, we concluded that the final model should ideally contain no more than 3–4 predictors of the investigated outcome (technical failure).

Six candidate predictors were entered into the multivariable model: sex, age, diabetes mellitus, hypertension, GLASS FP grade ≥ 2, and tibioperoneal trunk (TPT) occlusion. Sex, age, and diabetes mellitus were selected a priori, whereas hypertension, GLASS FP grade ≥ 2, and TPT occlusion were selected based on the univariable results and observed failure rates. This corresponds to 35/6 = 5.8 events per predictor, which is below the conventional threshold of 10; the implications of this are addressed in Section 5. Sex and age were included as basic demographic characteristics; hypertension was included because the proportion of patients with this condition was significantly lower among those with technical failure than among the others; and diabetes mellitus was included as the variable of primary interest, since patients’ exposure to this condition is the subject of the present study. GLASS FP grade ≥ 2 was chosen among the four possible thresholds based on the failure rates recorded by grade: FP 0 → 12.5%, FP 1 → 11.1%, FP 2 → 33.3%, FP 3 → 53.3%, FP 4 → 61.5%. Grades 0 and 1 are therefore almost identical, and the marked increase in the failure rate occurs at the transition from grade 1 to grade 2; furthermore, the ≥3 and =4 thresholds yield odds ratios like that of the ≥2 threshold. In turn, TPT occlusion coincides with GLASS IP grade 4 and is associated with a high OR of 4.089, the proportion of patients with TPT occlusion being significantly higher among those with technical failure than among those with technical success.

The predictors left out of the model were therefore: the large majority of the comorbidities, the WIfI stage and the FA (fibular artery) lesion grades (because no statistically significant differences were recorded between patients with technical success and those with technical failure); the SFA/POP/ATA/PTA/TPT lesion grades (because they are the components from which the GLASS FP and IP grades are derived, i.e., a situation of structural multicollinearity); the overall GLASS stage (because it is defined by the combination of FP and IP grades); and the presence of stents and of stenotic plaques (because their inverse association with failure reflects the outcome of the procedure rather than pre-procedural anatomy, since a stent can only be deployed after successful lesion crossing and their inclusion would introduce reverse causation).

The resulting model showed adequate calibration (Hosmer–Lemeshow χ^2^ = 5.575, df = 8, *p* = 0.695) and was statistically significant (omnibus likelihood-ratio χ^2^ = 35.409, df = 6, *p* < 0.001); it explained 33.7% of the variance in technical outcome (Nagelkerke R^2^). No multicollinearity problems were identified (tolerance 0.81–0.99; VIF 1.01–1.23).

Of the six candidate predictors entered in the model, those identified as significantly associated with technical failure were GLASS FP grade ≥ 2 (adjusted OR 8.068, 95% CI 3.018–21.562; *p* < 0.001) and tibioperoneal trunk occlusion (adjusted OR 4.874, 95% CI 1.271–18.687; *p* = 0.021), while the diagnosis of hypertension retained its inverse association with technical failure (adjusted OR 0.205, 95% CI 0.066–0.641; *p* = 0.006). Male sex, age and the diagnosis of diabetes mellitus were not associated with technical failure (Table 5, Figure 3). Bootstrap BCa confidence intervals confirmed the direction and statistical significance of the three identified associations (GLASS FP ≥ 2, tibioperoneal trunk occlusion and hypertension); the intervals for the two anatomical predictors were considerably wider than the asymptotic ones (Table 5), reflecting the limited number of events.

We also evaluated the multivariable model’s discriminatory performance using Receiver Operating Characteristic (ROC) analysis (Figure 4). The model successfully separated the case distributions, with an AUC of 0.820 (95% CI, 0.744–0.897; *p* < 0.001), indicating good discrimination. When the same model was applied within subgroups, the AUC was 0.881 (95% CI, 0.767–0.995; *p* < 0.001) in NDM patients and 0.772 (95% CI, 0.668–0.876; *p* < 0.001) in DM patients. The two ROC curves were not formally compared; the confidence intervals overlapped, and the estimates were imprecise given the small number of events per subgroup (14 and 21, respectively). These subgroup results should therefore be regarded as descriptive.

Clinical outcomes according to diabetic status are summarized in Table 6. Technical success rates were similar between groups (72.5% vs. 75.3%, *p* = 0.723). Major amputation and mortality were numerically higher in diabetic patients, although these differences did not reach statistical significance.

## 4. Discussions

The main finding of this study is that anatomical complexity was strongly associated with immediate technical outcome in infrainguinal CLTI. Technical success decreased progressively with increasing GLASS stage, with the strongest effect observed in the femoropopliteal segment. In multivariable analysis, GLASS FP ≥ 2 and tibioperoneal trunk occlusion were independently associated with technical failure, while hypertension showed an unexpected inverse association with failure. We detected no independent association between diabetes mellitus and immediate technical outcome in the present cohort. Similar findings have been reported in previous GLASS validation studies, in which increasing anatomical complexity was associated with progressively lower technical success and higher rates of immediate technical failure [10,11]. These findings are consistent with the original concept of GLASS, introduced in the Global Vascular Guidelines as a vascular map designed to guide revascularization planning in CLTI [1]. GLASS was developed not only to describe disease burden but to estimate the feasibility and durability of a selected target arterial pathway. In this context, our results support the potential value of GLASS for preprocedural assessment of anatomical complexity, particularly when anticipating lesions that may be difficult to cross and require alternative techniques.

In a systematic review and meta-analysis including more than 2200 patients and approximately 2500 limbs with CLTI, Shirasu et al. reported a progressive increase in technical failure across GLASS stages, with significantly higher failure rates observed in advanced disease [10]. Bontinis et al., in a meta-analysis of 11 studies including nearly 1800 patients and 1975 limbs, showed that GLASS stage III was associated with an almost fourfold higher risk of immediate technical failure compared with stages I-II [11]. Additional studies, including those by Liao et al., showed that higher GLASS stages were associated with a significant decline in 1-year amputation-free survival. At the same time, advanced infrapopliteal GLASS grades independently predicted the need for reintervention and poorer limb outcomes [20]. Recently, data published by Shahat et al. confirmed this relationship in both short- and mid-term follow-up after endovascular treatment [21]. In a study published this year, Melo et al. reported that increasing GLASS stage was associated with lower immediate technical success and a higher risk of limb-based patency failure. In patients with GLASS stage III, endovascular treatment was associated with a higher incidence of major adverse limb events than open bypass [22]. Our findings add to the growing body of evidence supporting the clinical applicability of GLASS in contemporary endovascular practice and underscore the need for further validation in different clinical settings. This need for continued validation and refinement has also been emphasized in the Global Vascular Guidelines, which acknowledge that GLASS requires further evaluation across different patient populations and healthcare environments [1]. To this end, the recently initiated prospective, international, multicenter PROMOTE-GLASS study aims to further evaluate the validity and reliability of the GLASS classification in predicting technical success, limb-based patency, major adverse limb events, and overall survival in patients with chronic limb-threatening ischemia undergoing endovascular treatment [23].

A key observation of the present study is the strong predictive value of the femoropopliteal component of GLASS. Proximal lesion complexity, including plaque cap morphology as described in the CTOP classification, is a major determinant of successful lesion crossing and procedural completion [3]. In clinical practice, long femoropopliteal lesions (>20 cm total length), chronic total occlusions, severe calcification, and choice of support catheters frequently dictate whether a lesion can be crossed and treated. This likely explains the strong performance of GLASS FP in our cohort. Our findings are directionally consistent with those of Meecham et al., who reported lower immediate technical success with higher GLASS stages in femoropopliteal endovascular interventions (97.5% in stage 1 vs. 84.0% in stage 3; *p* = 0.029) [24]. However, the absolute success rates in our cohort were lower (86.3% in stage I and 53.1% in stage III), and the gradient across stages was steeper. Meecham et al. also emphasized that, although GLASS stratifies anatomical complexity, further refinement may be needed to improve its clinical utility, particularly by incorporating inframalleolar disease and pedal runoff [24]. The infrapopliteal component showed a more nuanced behavior. GLASS IP was associated with technical outcome in the overall cohort, but this association was not significant among patients with diabetes. This may reflect the predominance of distal small-vessel involvement in diabetic CLTI, including below-the-ankle disease, a pattern described as small artery disease and below-ankle disease by Ferraresi et al. [8]. This information should not be interpreted as a lack of importance of distal disease. Revascularization often relies on a single patent vessel despite extensive distal occlusive disease, a pattern that GLASS IP grading does not adequately capture. Hata et al. demonstrated that GLASS retains predictive value in isolated below-the-knee lesions, particularly when infrapopliteal and inframalleolar disease coexist [14]. Similarly, Matsuura et al. reported that infrapopliteal GLASS staging is associated with wound healing and pain relief following partial revascularization [25]. These observations suggest that distal disease remains clinically relevant, but its predictive value may depend on additional factors, such as runoff quality, arterial calcification, and tissue loss.

The importance of distal and inframalleolar anatomy has been further emphasized by studies evaluating the GLASS inframalleolar modifier. Morisaki et al. demonstrated that the inframalleolar modifier is associated with limb salvage and wound healing following infrainguinal endovascular revascularization [15]. Iwata et al. highlighted the role of the GLASS P2 modifier in identifying a true “no-option” anatomical pattern in CLTI [16]. Global or segmental GLASS staging may benefit from additional stratification in patients with advanced distal disease, particularly in diabetic populations.

The predictive value of GLASS may vary depending on the revascularization strategy. Soares et al. found no significant association between GLASS stage and clinical outcomes or patency following distal bypass using autogenous vein grafts, suggesting that anatomical staging may be less predictive in the context of open surgical revascularization [26].

An important finding of the present study is that we detected no independent association between diabetes mellitus and technical failure in this cohort. Although diabetes is strongly associated with more diffuse distal arterial disease, our findings suggest that anatomical lesion characteristics were more closely associated with immediate procedural outcome in the present analysis [27]. Diabetes is known to influence the anatomical pattern of disease; in our cohort, however, immediate procedural outcome was more closely associated with the anatomical characteristics and the difficulty in passing the lesion that the operator must address (e.g., subintimal recanalization, re-entry devices). In this context, lesion morphology may also guide the choice of crossing strategy, as reflected in approaches such as the CTOP classification, where increasing complexity often necessitates planned retrograde or combined techniques obtained via distal tibial or pedal arteries (e.g., posterior tibial or dorsalis pedis), depending on runoff and target vessel patency, rather than late bailout conversion following antegrade failure [28]. This distinction is clinically relevant, as diabetes alone should not preclude an endovascular-first approach when the anatomical target is technically feasible. While technical success rates were similar between diabetic and non-diabetic patients, major amputation was numerically more frequent in the diabetic subgroup, although the difference was not statistically significant. Endovascular revascularization primarily restores macrocirculatory flow; however, limb salvage depends on a broader set of factors, including wound severity, infection, neuropathy, and microcirculatory function. This likely explains the dissociation observed in our study between technical success and clinical outcomes. Although the present study focused on immediate technical success, procedural success should be regarded as only one component of treatment effectiveness in patients with CLTI. Restoration of inline flow is an important prerequisite for limb salvage, but it does not necessarily translate into favorable long-term outcomes such as wound healing, freedom from reintervention, or overall survival. Recent data from the BEST-CLI trial showed that endovascular technical failure occurred in 16% of procedures and was most related to inability to cross the lesion (82%). Technical failure was associated with more complex multilevel occlusive disease, proximal superficial femoral artery occlusion, and a markedly higher rate of major adverse limb events at 3 years (81% vs. 29%) [29]. Campbell et al. further reported that patients with technical failure more frequently presented with long chronic total occlusions and severe calcification [30]. Therefore, future prospective studies with longer follow-up are needed to determine whether the anatomical predictors identified in the present analysis retain prognostic value beyond the procedural setting.

The multivariable analysis further supports the predominant role of anatomical complexity in immediate endovascular technical outcome. After simultaneous adjustment for sex, age, diabetes mellitus, hypertension, GLASS FP grade ≥ 2, and tibioperoneal trunk occlusion, both GLASS FP grade ≥ 2 and tibioperoneal trunk occlusion remained independently associated with technical failure. These associations were substantial, with adjusted odds ratios of 8.068 and 4.874, respectively. These findings are clinically plausible, as increasing femoropopliteal complexity reflects a more challenging target arterial pathway, whereas tibioperoneal trunk occlusion represents a particularly difficult pattern of distal disease for successful lesion crossing and restoration of inline flow. This finding does not exclude a clinically relevant influence of diabetes on the overall course of CLTI, but indicates that, within the present cohort and for the specific endpoint of immediate procedural success, diabetes mellitus was not independently associated with technical failure after adjustment for clinical and anatomical variables. An unexpected finding was the inverse association between hypertension and technical failure, which persisted after multivariable adjustment (adjusted OR 0.205, 95% CI 0.066–0.641). This result should not be interpreted as evidence of a protective effect of hypertension. The present data do not support a clear anatomical or procedural mechanism, and the association may reflect cohort-specific characteristics, residual confounding, or model instability related to the limited number of technical failures. In contrast, the associations observed for GLASS FP ≥ 2 and tibioperoneal trunk occlusion were consistent with the anatomical patterns identified in the univariable analyses and remained statistically significant after adjustment. Bootstrap resampling confirmed the direction and statistical significance of the associations with GLASS FP ≥ 2, tibioperoneal trunk occlusion, and hypertension, although the wider bootstrap confidence intervals for the anatomical predictors emphasize the uncertainty associated with the limited number of events. Accordingly, these results support the relevance of anatomical complexity rather than providing definitive effect-size estimates.

While anatomical complexity strongly influences procedural feasibility, limb-related outcomes are driven by clinical severity, as reflected in WIfI-based risk models [31,32]. In this context, hemodynamic assessment also remains essential, as pressure-based thresholds provide additional insight into ischemic burden beyond anatomical classification [33]. Supporting this concept, Chen et al. demonstrated that WIfI wound grade and clinical stage independently predict major adverse limb events and reduced amputation-free survival after endovascular treatment [22]. Similarly, Matsuura et al. showed that infrapopliteal GLASS grading is associated with wound healing following partial revascularization, highlighting the clinical relevance of distal disease beyond its impact on technical success [25]. Together, these findings support a complementary role for GLASS and WIfI in the evaluation of patients with CLTI.

## 5. Limitations

The relatively small sample size and the single-center retrospective design limit the generalizability of the findings. Accordingly, the observed technical success rate should be interpreted within the selected population of patients undergoing infrainguinal endovascular treatment and may not be applicable to the broader CLTI population, including patients undergoing primary bypass surgery or those with aortoiliac disease, and the reported associations require confirmation in larger prospective multicenter studies. As the study period corresponded to the early phase of the institutional endovascular program, a potential learning-curve effect on technical outcomes cannot be excluded. The ‘easiest-first’ target-vessel strategy may also have introduced some degree of operator-related selection bias. Several variables that may influence technical success, such as HbA1c, smoking status, formal calcification grading, lesion length, chronic total occlusion burden, and formal runoff assessment, were not available as separate variables, although lesion length and occlusion are partly reflected in the GLASS segmental grades. Residual confounding therefore cannot be excluded, and the absence of an association with diabetes should be interpreted considering the limited sample size. In addition, the primary endpoint was technical success, and long-term outcomes such as patency and limb salvage were not evaluated.

GLASS staging was performed retrospectively by a single experienced vascular specialist, and interobserver variability was not assessed. Since GLASS is an anatomical classification that requires interpretation of angiographic findings, future prospective studies should include independent observers and formal agreement analyses to further evaluate its reproducibility. Previous studies have shown that standardized GLASS assessment can achieve good interobserver agreement, supporting its use in clinical practice [7].

A number of methodological limitations related to the statistical analysis should be acknowledged. With only 35 technical failures, the number of events per candidate variable in the multivariable model (5.8) was below the conventional threshold of 10, so overfitting cannot be ruled out. All candidate variables were entered simultaneously (Enter method) without automated variable selection; however, the choice of candidate predictors was partly exploratory. We selected hypertension, tibioperoneal trunk occlusion, and the GLASS FP ≥ 2 threshold based on univariable results and observed failure rates rather than prespecification, although alternative cut-offs (≥3, =4) yielded similar associations. Bootstrap resampling retained the direction of the associations but yielded wide confidence intervals (e.g., 2.4–115 for GLASS FP ≥ 2), reflecting the limited number of events. The model was not externally validated, so its performance in other populations is unknown. The inverse association with hypertension was unexpected and may reflect residual confounding. The multiple subgroup and threshold analyses were exploratory, and no correction for multiplicity was applied. Finally, although the observed AUC of 0.820 indicates good discrimination, the model should be regarded as hypothesis-generating rather than a validated prediction tool.

Despite these limitations, the inclusion of real-world CLTI patients undergoing endovascular treatment reflects routine clinical practice and may enhance the clinical applicability of the findings, particularly by capturing the spectrum of anatomical complexity and comorbidity encountered in everyday endovascular practice.

## 6. Conclusions

Our findings suggest that immediate technical success after infrainguinal endovascular treatment is associated with anatomical complexity, while this cohort showed no independent association between diabetes and immediate technical outcome. Increasing overall GLASS stage was associated with a progressive decline in technical success, while GLASS-derived femoropopliteal complexity and tibioperoneal trunk involvement emerged as independent predictors of technical failure. Identifying anatomical predictors of technical failure may help anticipate difficult lesion crossing and guide timely transition to alternative revascularization strategies when endovascular attempts are unlikely to succeed. These findings support the clinical usefulness of the GLASS classification for preprocedural planning and warrant further validation in larger prospective multicenter studies.

## Figures and Tables

**Figure 1:**
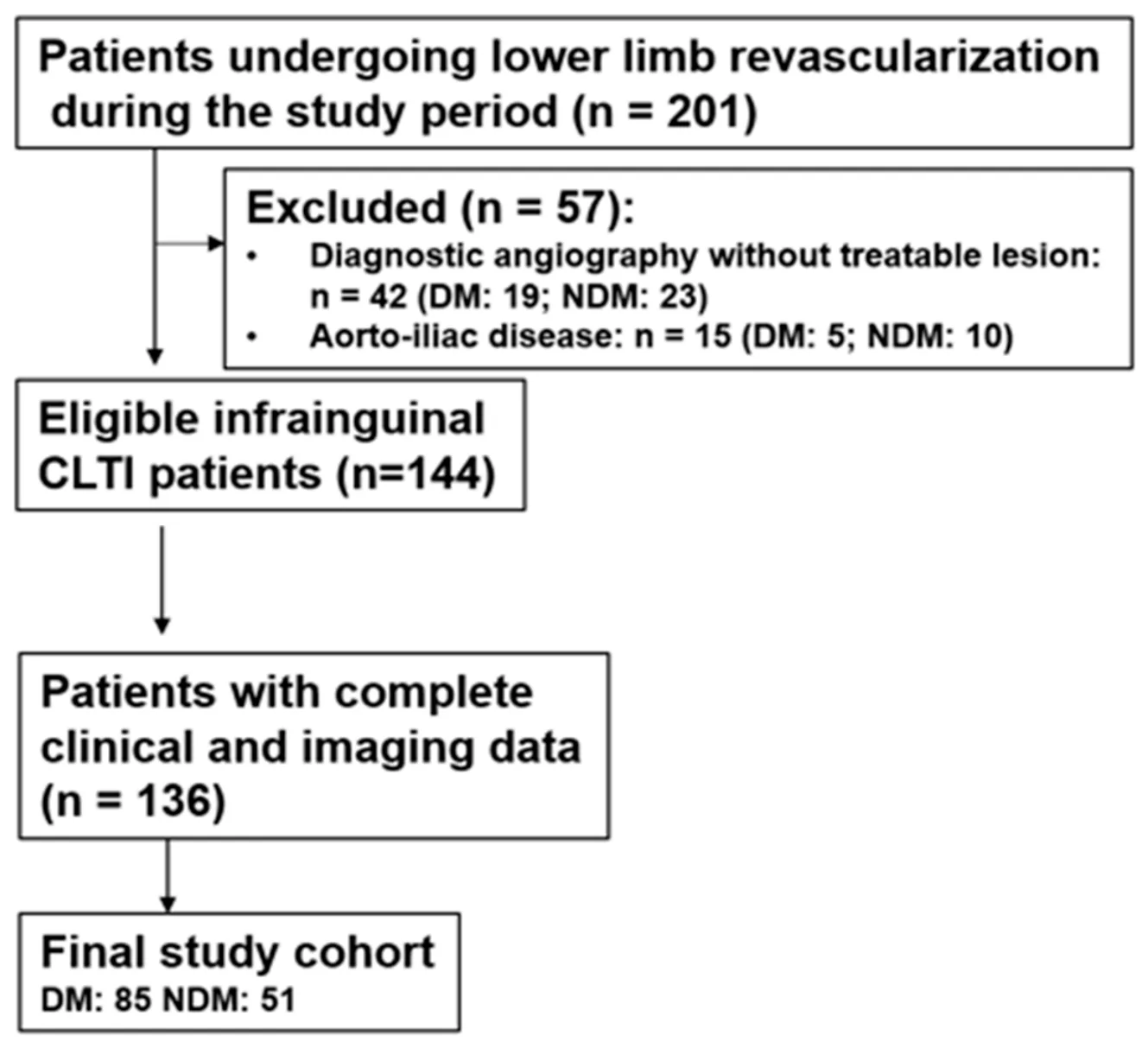
Flowchart of patient selection. The final study cohort comprised 136 patients, corresponding to 136 treated limbs and 136 endovascular procedures. No bilateral or repeated procedures were included.

**Figure 3:**
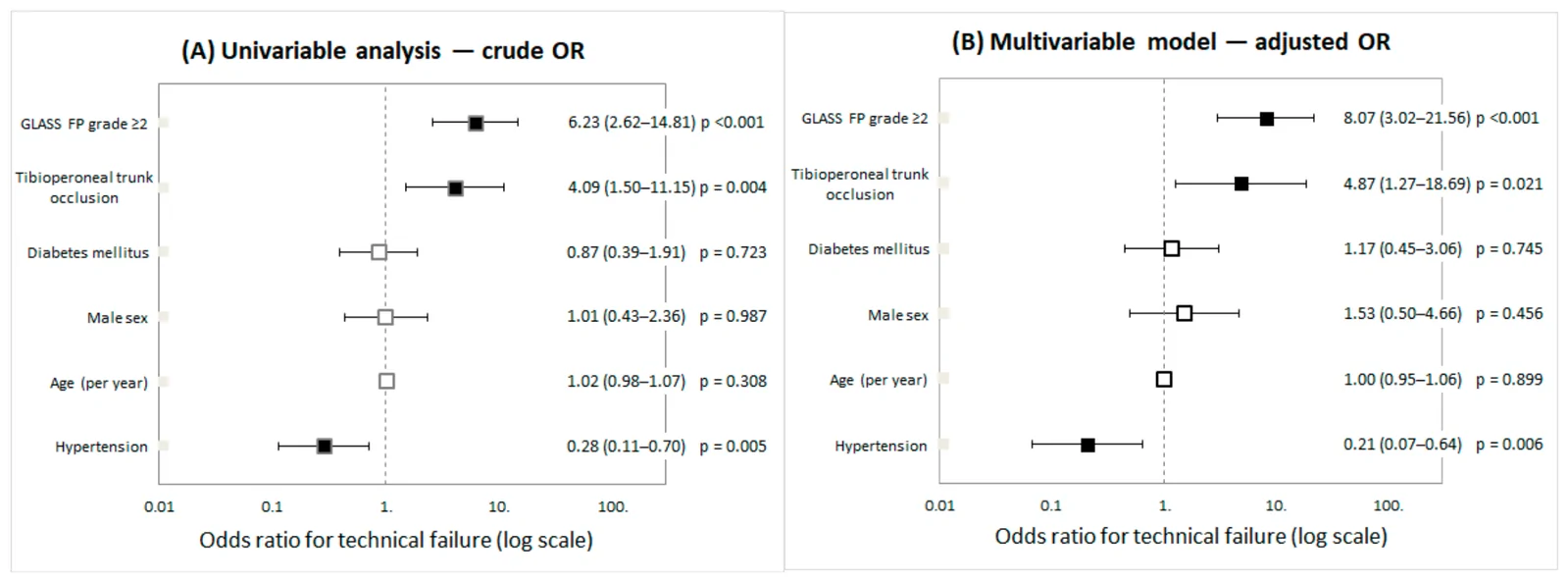
Forest plots of the odds ratios (OR) for immediate technical failure. (**A**) Crude ORs from univariable analysis; (**B**) adjusted ORs from the six-variable multivariable logistic regression model. Squares indicate point estimates (filled: *p* < 0.05; open: *p* ≥ 0.05) and horizontal lines the 95% confidence intervals, plotted on a logarithmic scale; the dashed line marks the line of no effect (OR = 1).

**Figure 4:**
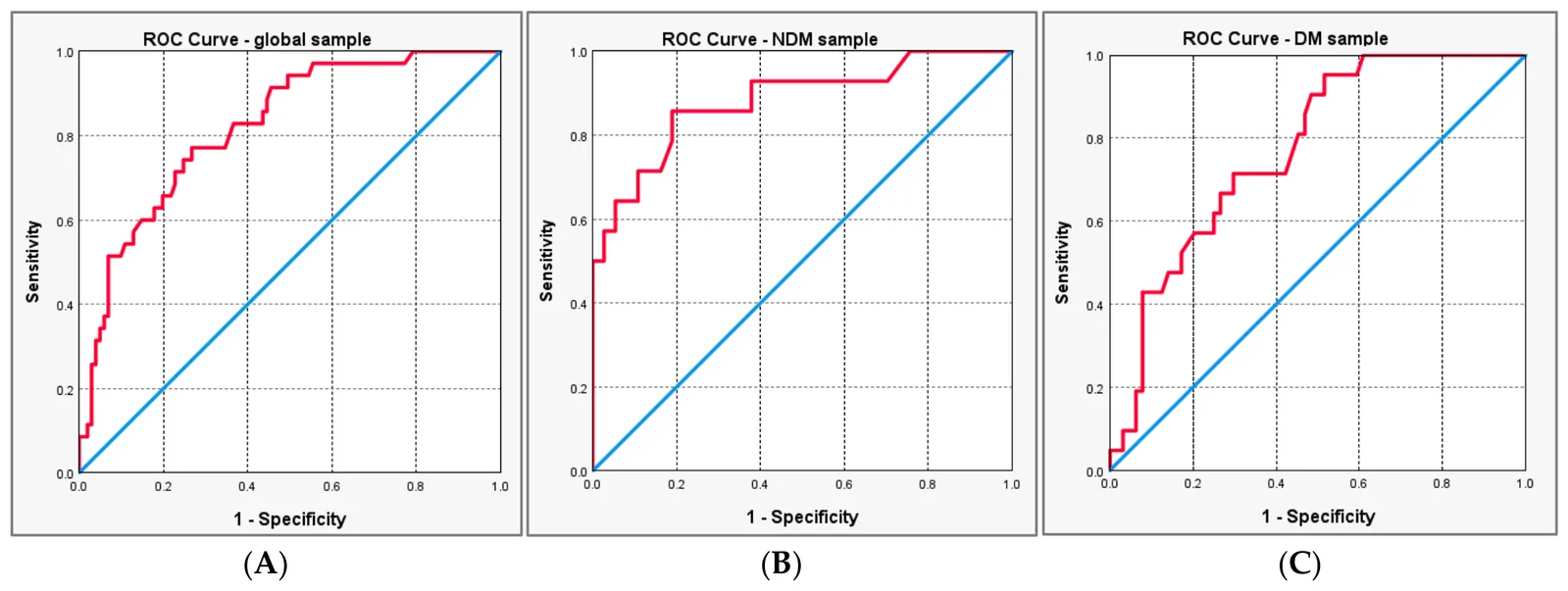
Receiver operating characteristic (ROC) curves of the multivariable model predicting technical failure. (**A**) Overall cohort (AUC = 0.820, 95% CI 0.744–0.897, *p* < 0.001). (**B**) Non-diabetic patients (AUC = 0.881, 95% CI 0.767–0.995, *p* < 0.001). (**C**) Diabetic patients (AUC = 0.772, 95% CI 0.668–0.876, *p* < 0.001). The red line represents the ROC curve of the predictive model, while the blue diagonal line represents the reference line corresponding to random classification (AUC = 0.5).

**Table 5:** Multivariable logistic regression analysis predicting technical failure.

Variable	Adjusted OR (95% CI)	*p*-Value	Bootstrap BCa 95% CI
Male sex	1.528 (0.501–4.660)	0.456	0.463–8.109
Age (per year)	1.003 (0.954–1.055)	0.899	0.952–1.066
Hypertension	0.205 (0.066–0.641)	0.006	0.046–0.611
Diabetes mellitus	1.172 (0.450–3.056)	0.745	0.400–3.695
GLASS FP ≥ 2 (vs. 0–1)	8.068 (3.018–21.562)	<0.001	2.411–115.0
Tibio-peroneal trunk occlusion	4.874 (1.271–18.687)	0.021 *	1.062–33.55

* Odds ratios (OR) with 95% confidence intervals (CI) are presented. Abbreviations: GLASS, Global Limb Anatomic Staging System; FP, femoropopliteal.

**Table 6:** Clinical outcomes according to diabetic status.

Outcome	Non-Diabetic (*n* = 51)	Diabetic (*n* = 85)	*p*-Value
Technical success	37 (72.5%)	64 (75.3%)	0.723
Technical failure	14 (27.5%)	21 (24.7%)	
Surgical bypass	6 (11.8%)	10 (11.8%)	1.000
Major amputation	2 (3.9%)	8 (9.4%)	0.320
Mortality	1 (2.0%)	3 (3.5%)	1.000

Values are presented as a number (%). Comparisons between groups were performed using the Pearson chi-square test or Fisher’s exact test (in case of Major amputation and Mortality), as appropriate. No statistically significant differences were observed between diabetic and non-diabetic patients.

## Data Availability

The original contributions presented in this study are included in the article. Further inquiries can be directed to the corresponding authors.

## Abbreviations

The following abbreviations are used in this manuscript:

CLTI	Chronic limb-threatening ischemia
GLASS	Global Limb Anatomic Staging System
ROC	Receiver operating characteristic
AUC	Area under the curve
OR	Odds ratio
CI	Confidence interval
WIfI	Wound, Ischemia, and Foot Infection
CTOP	Chronic Total Occlusion Crossing Approach based on Plaque Cap Morphology
